# Immunotherapy with FBTA05 (Bi20), a trifunctional bispecific anti-CD3 x anti-CD20 antibody and donor lymphocyte infusion (DLI) in relapsed or refractory B-cell lymphoma after allogeneic stem cell transplantation: study protocol of an investigator-driven, open-label, non-randomized, uncontrolled, dose-escalating Phase I/II-trial

**DOI:** 10.1186/1479-5876-11-160

**Published:** 2013-07-02

**Authors:** Raymund Buhmann, Stanglmaier Michael, Hess Juergen, Lindhofer Horst, Christian Peschel, Hans-Jochem Kolb

**Affiliations:** 1 Department of Medicine III, Klinikum der Universitaet Muenchen, Grosshadern, Marchioninistrasse 15, Munich 81377, Germany; 2 Helmholtz Center Munich, German Research Center for Environmental Health, Marchioninistrasse 25, Munich 81377, Germany; 3 Department of Medicine III, Klinikum rechts der Isar, Technische Universitaet Muenchen, Ismaningerstrasse 22, Munich 81675, Germany; 4 TRION Research GmbH, Am Klopferspitz 19, Martinsried 82152, Germany; 5 TRION Pharma GmbH, Frankfurter Ring 193a, Munich 80807, Germany

**Keywords:** B Cell Malignancies, Allogeneic Transplantation, Donor Lymphocyte Infusion, Immunotherapy, Trifunctional Bispecific Antibody (trAb)

## Abstract

**Background:**

Patients with B cell malignancies refractory to allogeneic stem cell transplantation (SCT) can be treated by subsequent immunotherapy with donor lymphocyte infusions (DLI). But unlike myeloid leukemia, B cell leukemia and lymphoma are less sensitive to allogeneic adoptive immunotherapy. Moreover, the beneficial graft-versus-lymphoma (GVL) effect may be associated with moderate to severe graft-versus-host disease (GVHD). Thus, novel therapeutic approaches augmenting the anti-tumor efficacy of DLI and dissociating the GVL effect from GVHD are needed. The anti-CD20 x anti-CD3 trifunctional bispecific antibody (trAb) FBTA05 may improve the targeting of tumor cells by redirecting immune allogeneic effector cells while reducing the risk of undesirable reactivity against normal host cells. Hence, FBTA05 may maximize GVL effects by simultaneously decreasing the incidence and severity of GVHD.

**Methods/Design:**

Based on this underlying treatment concept and on promising data taken from preclinical results and a small pilot study, an open-label, non-randomized, uncontrolled, dose-escalating phase I/II-study is conducted to evaluate safety and preliminary efficacy of the investigational antibody FBTA05 in combination with DLI for patients suffering from rituximab- and/or alemtuzumab-refractory, CD20-positive low- or high-grade lymphoma after allogeneic SCT. During the first trial phase with emphasis on dose escalation a maximum of 24 patients distributed into 4 cohorts will be enrolled. For the evaluation of preliminary efficacy data a maximum of 12 patients (6 patients with low-grade lymphoma and/or Chronic Lymphocytic Leukemia (CLL) / 6 patients with high-grade or aggressive lymphoma) will attend the second phase of this clinical trial.

**Discussion:**

Promising data (e.g. induction of cellular immunity; GVL predominance over GVHD; achievement of partial or complete responses; prolongation of time-to-progression) obtained from this phase I/II trial would represent the first milestone in the clinical evaluation of a novel immunotherapeutic concept for treatment-resistant low- and high-grade lymphoma and NHL patients in relapse.

**Trial registration:**

NCT01138579

## Background

### B cell malignancies

Approximately half of the patients with aggressive non-Hodgkin's lymphomas (NHL) and the majority of patients with low-grade lymphomas cannot be cured by conventional therapeutic treatment regimes. Intensive chemotherapy followed by stem cell support has improved survival of patients with chemotherapy sensitive lymphoma. The majority of these patients, however, will still relapse, even after high-dose therapy and subsequently have limited therapeutic options. Although allogeneic stem cell transplantation (allo-SCT) can induce long-term remission due to a graft versus lymphoma (GVL) reaction, this occurs predominantly in a highly selected population of indolent lymphoma patients. A GVL reaction is less pronounced in patients with aggressive lymphoma, resulting in a high relapse rate [1-7]. In patients not eligible for allogeneic stem cell transplantation, palliative treatment is restricted by resistance to chemotherapy and complications of infection. Therefore, new therapeutic strategies with improved anti-tumor efficacy need to be developed for such patients.

Immunotherapies with monoclonal antibodies (mABs) directed against the CD20 antigen have previously been shown to be highly effective. Rituximab (Rituxan; Genentech Inc, South San Francisco, CA, and Biogen IDEC Inc, Cambridge, MA), an immunoglobulin gamma (IgG) 1 chimeric mAb, induced overall response rates (ORR) up to 50% in patients with relapsed or refractory low-grade B cell lymphoma [8,9]. In first line-treatment, rituximab induces response rates up to 75% in patients with follicular or low-grade NHL [10]. But despite these encouraging results, there are still numerous patients who do not respond or finally relapse [11].

Efforts to further increase the therapeutic efficacy of antibodies resulted in the development of trAb FBTA05 which mediates effective tumor cell cytotoxicity even at low CD20 expression levels, and probably even more importantly, induces a lasting anti-tumor immunity [12-15].

### Compound review

FBTA05 is a heterologous antibody, composed of two potent heavy chain subclasses, a mouse IgG2a and a rat IgG2b chain, each with their respective light chains. It therefore possesses two specific and one functional binding site. FBTA05 binds with one binding arm to the CD3 antigen on T cells and with the second binding arm to the CD20 antigen, which is expressed exclusively on normal and malignant B cells, but not on hematological precursor cells or other human cell types. In addition, the third functional site within the hybrid Fc region binds to Fcγ receptor type I, IIa and III which are expressed by accessory cells (e.g., macrophages, dendritic cells, natural killer cells) of the immune system. In this “tri-cell complex”, an important “crosstalk” between T cells and accessory cells can occur, which includes co-stimulatory signals necessary for a physiological T cell activation cascade. The simultaneous activation of different immune cells at the tumor site results in efficient killing of tumor cells by several complementary mechanisms (e.g. release of cytokines, perforin-mediated lysis and phagocytosis). The antibody-mediated phagocytosis of tumor cells by accessory cells (macrophages and dendritic cells) is believed to result in the processing of tumor antigens and presentation on the surface of these cells. Since this can result in polyclonal humoral and cellular immune responses [13,14], a T cell response even against unknown, tumor-associated peptides may be induced.

**Figure 1:**
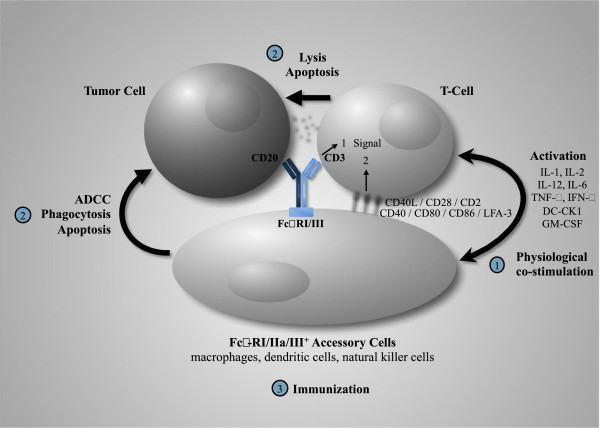
**FBTA05 (anti-CD3 x anti-CD20 trAb) – mode of action.** In a MHC-independent manner polyclonal T cells are redirected to and activated at tumor cells by trAb-mediated recognition of CD3 and tumor-associated antigens (TAAs) such as CD20. At the same time or subsequently FcγR-positive accessory cells such as monocytes/macrophages, dendritic cells (DCs) or natural killer cells interact with the Fc part of trAbs. Through this cellular crosstalk all participating immune cells are strongly activated. Hence, T cells receive a second co-stimulatory signal, while accessory immune cells are stimulated via FcγR crosslinking which leads to the release of proinflammatory cytokines. Thus, tumor cells are effectively eliminated by a concerted attack of cytotoxic T cells and accessory immune cells using different killer mechanisms such as ADCC, phagocytosis, or perforin/granzyme-mediated lysis and apoptosis induction. Finally, T cell proliferation occurs as does phagocytosis of necrotic/apoptotic tumor particles which are then processed, and presented by stimulated professional antigen presenting cells (i.e. DCs), an important prerequisite for the induction of long-lasting vaccination-like effects against tumors. ADCC (antibody dependent cellular cytotoxicity); CMC (complement-mediated cytotoxicity); DC-CK1 (Dendritic cell cytokine 1); IL (interleukin); LFA (leukocyte function-associated antigen); TNF-α (Tumor necrosis factor α).

The combined attack of various immune cells with different killing mechanisms induced by FBTA05 leads to a significant tumor cell elimination when compared to monospecific antibodies or conventional bispecific antibodies. In addition, the induction of a variety of effector mechanisms may result in a protective long-term anti-tumor immunity. The postulated mechanism of action of the trAb FBTA05 is shown in Figure 1.

### Previous trials and clinical data

Prior to the start of the current phase I/II dose escalating study, FBTA05 was administered in a compassionate use setting to patients with recurrent and refractory B cell malignancies following allo-SCT, and within a phase I/II trial (IV-A05-LL-01, EudraCT No: 2006-006694-24) to patients with relapsed or refractory CLL in an autologous setting.

In compassionate use a limited number of advanced cancer patients were treated with FBTA05. These, included 4 patients with refractory Chronic Lymphocytic Leukemia (CLL), 4 patients with refractory high-grade non-Hodgkin Lymphoma (HG-NHL) and 1 patient with refractory Acute Lymphocytic Leukemia (ALL) [16]. The treatment comprised escalating doses of FBTA05 with a range of 10–2,000 μg per single infusion. The maximum cumulative dosage administered per patient ranged between 130–13,610 μg of FBTA05. The duration of treatment was between 5–76 days. Following FBTA05 treatment, patients received DLI. Due to the nature of the different indications, the number of peripheral B cells prior to the start of FBTA05 infusions showed a large variation. An anti-tumor response (decrease of leukemic cells and shrinking of lymph nodes) was observed in 3 of 4 CLL patients. In 1 case of HG-NHL, a halt of progression for almost 4 months was observed. The effect of FBTA05 in the other patients was not evaluable due to other concomitant treatment or disease (fungal infection). Relapse finally occurred in all patients. The main side effects were restricted to fever, chills, bone pain and transient increase in γ-glutamyl transpeptidase (γGT). Only one case of GVHD (grade 3) was observed after FBTA05 and DLI and this was in the ALL patient. In context of the administration of the highest cumulative dose of FBTA05 (6,240 μg), a transient granulocytopenia was detected in a HG-NHL patient. The cytokine profile was characterized by a transient increase of IL-6, IL-8 and IL-10. Human anti-mouse antibodies (HAMAs) were not detectable. According to the current experience with the anti HER2/neu directed trAb ertumaxomab there is a correlation between the formation of HAMA and human anti-rat antibodies (HARAs), so HARA development in these patients would seem to be unlikely [17].

Due to the changes in the development program of the sponsor (Fresenius Biotech GmbH) the phase I/II dose escalating study (IV-A05-LL-01, EudraCT No: 2006-006694-24) was prematurely terminated after treatment of 3 patients in the first cohort (maximum dose 100 μg FBTA05). Thus, no maximum tolerated dose (MTD) could be established and only limited data on drug safety are available. In general, the intravenous infusion of FBTA05 was assessed to be safe and well tolerated, even at higher antibody doses. However, due to the limited number of patients treated, to variable disease progression in individual patients and to a variety of different concomitant treatments used, this information needs to be interpreted with caution.

### Rationale for performing the study

Antibody based therapies (rituximab, alemtuzumab), especially in combination with chemotherapeutic regimes, offer some advantages in patients with CLL and B cell non-Hodgkin lymphoma (NHL). Nevertheless, most patients finally relapse. Especially patients with diffuse large B cell lymphoma (DLBCL) have a very poor prognosis in case of early relapse after chemotherapy combined with rituximab. Therefore, there is an urgent need to further develop alternative treatment options whereby the CD20 antigen represents a solid target for innovative therapeutic approaches: (i) it is expressed on most B-cell lymphomas, (ii) it is only expressed on B cells, but not precursor cells or human tissues and (iii) it is not shed or secreted upon antibody binding.

The trAb FBTA05 provides a novel and unique approach for the targeted treatment of CD20 expressing neoplasias. The potency of FBTA05 has been demonstrated by efficient in vitro killing of human B cell tumor cells, as well as cells derived from CLL patients expressing only low levels of CD20 [12]. FBTA05 in combination with DLI has already been administered to patients in compassionate use prior to the start of the scheduled phase I/II study and assessed to be feasible, safe and well tolerated even at high antibody and T cell doses. Most importantly, some anti-tumor responses were observed in 3 CLL and 1 NHL patient [16].

It is well established that DLI, given as a single treatment is effective in hematological relapse post-SCT, but it is suggested that concerted treatment with additional drugs may further improve treatment outcome [18,19]. Thus, the combination of DLI and the anti-CD20 x anti-CD3 trAb FBTA05 might be a promising treatment option for patients with B cell malignancies refractory to allo-SCT. Since both, the desired GVL and the unwanted detrimental GVHD responses are mediated by T lymphocytes present in DLI preparations, FBTA05 has the capacity to enhance these GVL effects as autologous and allogeneic T cells are targeted to tumor sites. At the same time as GVL of allogeneic T lymphocytes is strengthened by FBTA05 engagement the risks for GVHD development are reduced. Therefore, by adding FBTA05 to the established allogeneic DLI treatment regime patients may even benefit more from this combined therapeutic approach. Furthermore, FBTA05 should be given prior to DLI administration to allow the already bound anti-CD20 x anti-CD3 trAbs FBTA05 to attract incoming allogeneic T lymphocytes directly to the CD20-expressing tumor cells. In other words, this treatment setting may favor GVL enhancement by simultaneously minimizing GVHD risks. This application sequence may be also important to control cytokine release due the increased number of CD3^+^ T target cells of FBTA05 in the blood circulation after DLI infusion. The opposite application order for FBTA05 and DLI would not mediate this positive effect of pre-coating tumor cells with trAb FBTA05 in vivo for attracting incoming allogeneic T lymphocytes. Moreover, preclinical and first clinical data confirmed this beneficial sequence for FBTA05 and DLI application.

## Methods/design

The present trial is an investigator-initiated, open-label, multi-center, non-randomized, uncontrolled, dose-escalating phase I/II study designed to evaluate the safety and efficacy of the investigational trAb FBTA05 in combination with DLI for treatment of relapsed or refractory disease in CD20 positive CLL / low-grade NHL or high-grade NHL after allogeneic transplantation.

### Primary and secondary objectives

The primary objective is the determination of the MTD of FBTA05 in phase I (dose-escalation part). In phase II (efficacy part), it is the preliminary evaluation of efficacy of a treatment schedule with FBTA05 and DLI in patients with CD20 positive either CLL / low-grade NHL or high-grade NHL after allo-SCT. The secondary objectives are the evaluation of safety and pharmacodynamics of FBTA05 in combination with DLI, the induction of cellular immunity in terms of GVL or GVHD, and the determination of further efficacy data in terms of time to progression (TTP), duration of response and clinical benefit.

### Primary and secondary endpoints

The primary endpoint in Phase I is the incidence of dose limiting toxicities (DLTs), in phase II the objective response rate. The secondary endpoints are safety and efficacy. Regarding safety, the recommended dose of FBTA05 in combination with DLI for further efficacy studies will be determined. Furthermore, the incidence of adverse events (AEs), the presence of HAMAs after FBTA05 application, the need to discontinue FBTA05 infusion, vital functions, the physical examination findings, laboratory parameters, concomitant medication and as pharmacodynamic endpoints the serum levels of cytokines will be recorded.

In terms of efficacy the clinical benefit rate, the duration of response, the time to progression, the overall survival, the ECOG performance status, the tumor specific response, lymphocyte subsets, activation signs and memory status of T cells will be analysed.

### Study design

**Figure 2:**
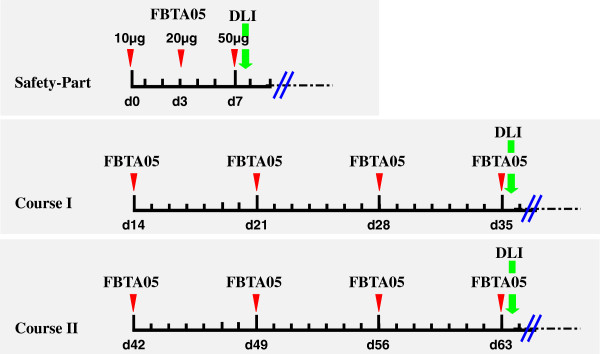
**Treatment schedule.** The FBTA05 treatment schedule consists of three parts: the drug induction part (safety part) and the dose maintenance / escalation parts (course I / II). Thereby the safety part is the same for each study patient, with FBTA05 applications of 10 μg on day 0, 20 μg on day 3 and 50 μg on day 7. FBTA05 dose adjustment for the subsequent treatment courses I and II, will be performed according to the respective cohort A-D (Table 1). Infusion of donor lymphocytes (DLI) will be dose-escalated (Table 3) and applied subsequent to the respective antibody infusions on day 7, day 35 and day 63 of the treatment schedule. The red arrows indicate the application of FBTA05, the green arrows the infusion of donor lymphocytes.

To evaluate differences in drug response to CLL/low grade NHL or high grade NHL, patients are enrolled into study cohorts according to their respective disease entity (either CLL / low-grade NHL or high-grade NHL). As shown in Figure 2, the complete treatment course consists of a drug induction part (safety part) with defined FBTA05 doses of 10 μg on day 0, 20 μg on day 3 and 50 μg on day 7, as well as a drug maintenance / escalation part (course I and II, see also Table 1). The final antibody infusion of each part is followed by a dose-escalated application of donor lymphocytes.

**Table 1:** Dose escalation of FBTA05

**FBT05**	**Day**	**Cohort A**	**Cohort B**	**Cohort C**	**Cohort D**
**Safety part**	**d0**	10 μg	10 μg	10 μg	10 μg
	**d3**	20 μg	20 μg	20 μg	20 μg
	**d7**	50 μg	50 μg	100 μg	200 μg
**FBT05**	**Day**	**Cohort A**	**Cohort B**	**Cohort C**	**Cohort D**
**Course I**	**d14**	50 μg	50 μg	100 μg	200 μg
	**d21**	50 μg	50 μg	100 μg	200 μg
	**d28**	50 μg	50 μg	100 μg	200 μg
	**d35**	50 μg	100 μg	200 μg	300 μg
**Course II**	**d42**	50 μg	100 μg	200 μg	300 μg
	**d49**	50 μg	100 μg	200 μg	300 μg
	**d56**	50 μg	100 μg	200 μg	300 μg
	**d63**	50 μg	100 μg	200 μg	300 μg

FBTA05 (anti-CD3 x anti-CD20 trAb).

Each cohort includes at least 2 patients. In a strict sequential order, treatment and dose escalation (cohort A-D, Table 1) is performed independently for each disease entity (either CLL / low-grade NHL or high-grade NHL). Thus, treatment of any subsequent patient 2 or 3 at a certain dose level will be started only after the safety part treatment of the previous patient of the same disease entity is completed, and the External Safety Board (ESB), acting as a Dose Steering Board (DSB), agrees to the enrolment of the next patient. The same dose escalation procedure will be applied throughout the study for all Cohorts (A-D). If one dose-limiting toxicity is seen, a total of 3 patients of the same disease entity must be treated at that dose level. If two dose-limiting toxicities are seen at any dose level, treatment for this disease entity is terminated and the MTD will be established at the next lower dosing level. If 2 of 3 patients of the same disease entity in Cohort A show dose-limiting toxicities, the MTD has been reached and lower dose levels may have to be investigated.

After the phase I part of the study an interim analysis will be performed. Based on the outcome of this analysis the recommended dose (RD) for the phase II part will be proposed by the ESB and the investigators on the basis of the MTD and any other relevant safety issues. The recommended dose will not exceed the maximal dose foreseen in the protocol approved by the competent authority. The report of the interim analysis with the recommended dose will be presented to the ethics committee and competent authority. A maximum of 6 patients per disease entity will receive treatment during phase II. If the last Cohort at MTD level in phase I already included 2 or 3 patients for CLL/low grade NHL, respectively high-grade NHL, an additional 4 or 3 patients per disease entity will be enrolled in phase II.

### Number of patients and eligibility criteria

There will be a maximum of 24 patients in the MTD evaluation part of phase I: 12 patients for CLL / low-grade NHL, 12 patients for high-grade NHL. There will be a maximum of 12 patients in phase II of the study: 6 patients for CLL / low-grade NHL, 6 patients for HG-NHL. A total of maximal 30 patients will be included in the study.

Patients of both genders, any ethnic origin and at least 18 years old are included in the study if they meet the criteria given in Table 2. All participating patients have to sign the written informed consent. Patients can be withdrawn from the study for medical reasons at any time and must be withdrawn in the event of a dose-limiting toxicity. Patients can recall their consent at any time without any negative implications for their further treatment.

**Table 2:** Eligibility criteria of the FBTA05 trial

	
**Inclusion criteria**	- confirmed CLL, low-grade NHL or high-grade NHL
	- CD20 positivity
	- relapsed or refractory disease > 60 days after allogeneic transplantation
	- adequate hematological, liver and kidney functions
	- platelet count ≥ 25 G/l
	- ECOG performance status ≤ 2
	- negative pregnancy test (adequate contraception during study in women of child bearing potential)
**Exclusion criteria**	- anti-CD20 or anti-T cell antibody treatment < 3 months before FBTA05 treatment
	- positivity for human anti-mouse antibodies
	- history of GVHD °III or °IV, or GVHD requiring steroid therapy with ≥ 10 mg / day
	- known hypersensitivity to recombinant murine or rat proteins
	- acute or uncontrolled chronic infections, viral infections at risk of reactivation (e.g. HCV, HBV, HIV)
	- patients unable or unwilling to comply fully with the protocol

*CLL* chronic lymphocytic leukemia, *NHL* Non-Hodgkin's lymphoma, *ECOG* Eastern Cooperative Oncology Group, *HAMA* human anti-mouse antibody, *GVHD* graft versus host disease, *HCV* hepatitis C virus, *HBV* hepatitis B virus, *HIV* human immunodeficiency virus.

### Drug formulation

The investigational drug FBTA05 is provided by the TRION Pharma GmbH (Munich, Germany) as a sterile, pyrogen-free, color-free and preservative-free solution for infusion. The concentrate contains 0.2 mg/ml antibody per 100mM sodium citrate buffer (pH 5.6), with 0.02% Tween 80. Depending on the dose level, FBTA05 is further diluted in 0.9% sodium chloride solution for i.v. infusion.

### Study treatment

FBTA05 is administered with a constant rate over 6 hours by intravenous (i.v.) infusion. To avoid infusion reactions typically occurring after i.v. antibody infusions, i.v. Paracetamol (1,000 mg) and i.v. Dimetinden (4 mg) are administered 30–60 minutes prior to the start of infusion. Three hours after the start of FBTA05 infusion, i.v. Paracetamol (500 – 1,000 mg) is repeated. Post-infusion, Paracetamol and Dimetinden are administered, as needed.

In phase I, each patient (cohort A – D) will undergo the same safety part and receive induction doses of FBTA05 on day 0 (10 μg), day 3 (20 μg) and day 7 (50 μg). During the maintenance part, FBTA05 applications are scheduled for course I on day 14 (± 1 day), 21 (± 1 day), 28 (± 1 day) and 35 (± 1 day), for course II on day 42 (± 1 day), 49(± 1 day), 56 (± 1 day) and 63 (± 1 day). Thereby dose escalation of FBTA05 will be performed according to the respective Cohort A – D (Table 1).

Donor lymphocyte infusion is scheduled in each cohort at the end of the safety part (day 7), as well as at the end of course I (day 35) and course II (day 63). The numbers of infused T cells are escalated according to the respective preparative regimen applied for allo-SCT as shown in Table 3. DLI will not be performed in case the of GVHD or active infection at the time of DLI, or in the rare cases that DLI is not available for technical reasons. In this case antibody application will be continued as scheduled without DLI.

**Table 3:** Dose escalation of donor lymphocyte infusions (DLI)

**DLI**	**Haplo-identical SCT**	**HLA-identical SCT**
**d7**	5 ×10^5^ /kg CD3^+^ cells	1 ×10^6^ /kg CD3^+^ cells
**d35**	1 ×10^6^ /kg CD3^+^ cells	5 ×10^6^/kg CD3^+^ cells
**d63**	5 ×10^6^ /kg CD3^+^ cells	1 ×10^7^/kg CD3^+^ cells

*SCT* stem cell transplantation, *HLA* human leukocyte antigen.

In phase II the recommended dose will be applied according to the respective treatment schedule as determined in phase I.

### Study visits

Patients are required to complete screening procedures and 14 treatment visits (11 applications of FBTA05; 3 applications of DLI), so far as the dosage regimen is tolerated according to MTD assessments. Two weeks after the last infusion (week 12), patients will attend an end-of-study visit (EOS). In follow up, patients will attend 4 additional post-study follow-up visits (6, 9, 12 and 24 months after start of treatment). Patients enrolled in phase II will follow the identical screening, treatment and post-study follow-up schedule as for phase I.

### Safety management

An ESB, composed of three independent experienced clinical experts is responsible for the evaluation of the patients. Together with the investigators they decide, whether individual patients may continue the study, and whether or not dose escalation can be applied. The ESB is involved in the assessment and declaration of Serious Adverse Events (SAEs), Suspected Unexpected Serious Adverse Reactions (SUSARs) as well as the evaluation of dose-limiting toxicities (DLT). Moreover, based on the results of the interim analysis at the end of the phase I, the ESB together with the investigators will propose the recommended dose (RD) for phase II.

The condition for a DLT is fulfilled in the case of termination of treatment due to an adverse reaction. Thereby, the treatment schedule for a patient must be terminated if any toxicity grade ≥ 3, condition or adverse event (e.g. body temperature ≥ Grade 3 Common Toxicity Criteria (CTC) (40°C), systolic BP < 75 or > 210 mm Hg, pulse < 50 or > 150 bpm or a dyspnoea grade IV) does not normalize within 3 hours. Furthermore, treatment will be terminated in case of an anaphylactic reaction, including, but not limited to severe angioedema, severe bronchospasm, severe urticaria or anaphylactic shock which occurs during the infusion. An adverse reaction, including clinical conditions of CTC Grade ≥ 3 or clinically relevant laboratory abnormality of CTC Grade ≥ 3 which occurs after the infusion and does not resolve until the planned date of the next infusion will also be grounds for termination of treatment.

### Safety, laboratory and imaging assessments

To assure the safety of the study patients a substantial examination program is performed. The patients are under hospital surveillance at least 1 hour prior to infusion and for 24 hours after the start of infusion. Physical examinations are performed at all scheduled visits and vital parameters (e.g. blood pressure, pulse rate, respiratory rate, oxygen-saturation and body temperature) are monitored throughout the infusion time until 24 hours after start of infusion. Moreover, patients are evaluated with regard to clinical signs, symptoms (including AEs) and subjective well-being.

Laboratory parameters for safety analysis and immune monitoring are evaluated at every study visit. Moreover, during treatment, blood will be drawn prior to the start of infusion, at the end of the 6-hour drug infusion period and at 24 after the start of infusion. Blood chemistry parameters include sodium, potassium, calcium, chloride, bilirubin (total), aspartate aminotransferase (AST, or SGOT), alanine aminotransferase (ALT, or SGPT) gamma-glutamyltransferase (GGT), alkaline phosphastase, antithrombin III, albumin, creatinine, uric acid, lactate dehydrogenase, partial thromboplastin time (PTT), quick prothrombin time, fibrinogen and the C-reactive protein (CRP). Hematology parameters encompass hematocrit, hemoglobin (Hb), red blood cell (RBC) count, white blood cell (WBC) count, reticulocytes, platelets and differential WBC. An extensive immune monitoring program determines lymphocyte subsets (CD4, CD8 T cells, B cells, NK cells, monocytes) including effector, central memory as well as regulatory T cells and cytokine release profiles comprising interleukin-2 (IL-2), IL-4, IL-6, IL-8, IL-10, interferon-gamma (IFN-γ), and tumor necrosis factor-alpha (TNF-α). Experimental ELISPOT-analysis to evaluate the induction of tumor specific immune responses after treatment with FBTA05 is restricted to CLL samples.

Bone marrow biopsies are done before the start of treatment, moreover at the EOS and, 12 and 24 months after the start of therapy. Disseminated tumor cells will be determined by analyzing bone marrow aspirates taken from the iliac crest. Changes in the presence of disseminated tumor cells in the bone marrow will be assessed according to NCI criteria.

Tumor assessment via imaging techniques (sonography, compute tomography, magnetic resonance tomography or positron-emission tomography) is clinical practise for the advanced stage of disease and will be performed at the screening, end of study visit and follow up visits scheduled 6, 9, 12 and 24 month after onset of the study. Assessment will be done according to Response Evaluation Criteria in Solid Tumors (RECIST).

### Statistics

The study is exploratory and is not powered to address any predefined hypotheses. The safety analysis will be performed on the safety analysis set, which includes all patients who received at least one FBTA05 infusion. The efficacy analysis will be performed on both the safety analysis set, which includes all patients who received at least the first three infusions and the first DLI (safety part). Efficacy endpoints will be analyzed by use of appropriate descriptive techniques.

### Trial organization and administration

#### Funding

The FBTA05 trial is supported by the Bavarian Immunotherapy Network (BayImmuNet) and the Munich m4-Biotech Cluster. BayImmunet is funded by the Bavarian State Ministry of Sciences, Research and the Arts (Bayerisches Staatsministerium für Wissenschaft, Forschung und Kunst (STMWFK); F5121.7.1.1/14/3). The Munich m4-Biotech Cluster is supported by the German Ministry of Research and Education (Bundesministerium für Bildung und Forschung (BMBF); 01EX1021A). The investigational drug FBTA05 used in the present trial is provided by Trion Pharma GmbH (Munich, Germany). No financial support is given other than the funding mentioned above. There are no restrictions on publications. Industrial funders and trial management are independent.

#### Sponsor

Klinikum rechts der Isar (RdI), Technische Universitaet Muenchen (TUM).

### Study approval

Before start of the trial, the trial protocol, informed consent document and any other trial documents were submitted to the independent ethics committee and the regulatory authority (Paul-Ehrlich-Institute, PEI). Ethics approval was granted on 14 January 2010, PEI approval on 16 July 2010.

### Registration

The trial protocol was registered at http://www.clinicaltrials.gov and was given the number NCT01138579.

### Good clinical practice

The procedures set out in this trial protocol, pertaining to the conduct, evaluation and documentation of this trial, are designed to ensure that all persons involved in the trial abide by Good Clinical Practice [20] and the ethical principles described in the current revision of the Declaration of Helsinki [21]. The trial will be carried out in keeping with local legal and regulatory requirements.

## Discussion

The idea to redirect immunity via bispecific antibodies is currently one of the most compelling concepts in cancer treatment. In the meantime, various bispecific antibody formats have entered clinical trials indicating their enormous therapeutic potential [22-24].

In the present phase I/II trial, the anti-CD3 x anti-CD20 trAb FBTA05 is applied in combination with DLI in patients with CD20-positive CLL, low- and high-grade NHL relapsed or refractory after allo-SCT. The primary goal of this combined FBTA05/DLI treatment approach is to further improve an already successful allogeneic DLI regime for patients with resistant B cell malignancies who relapsed after allo-SCT [25,26]. In addition, Riechelmann and co-workers showed that opsonization of autologous effector cells (i.e. peripheral blood mononuclear cells) with anti-EpCAM x anti-CD3 trAb catumaxomab led to beneficial results (i.e. tolerability, safety and response) in patients with intractable recurrent head and neck squamous cell carcinomas [27]. Based on these findings and anticipating our own preliminary clinical data from a small pilot study [16], the planned treatment schedule is designed to enhance the targeting of tumor cells by allogeneic T lymphocytes while reducing the risk of undesirable GVHD reactivity against normal host cells. Hence, FBTA05 may maximize GVL effects by simultaneously decreasing the incidence and severity of GVHD as already shown in preclinical models [28,29]. Of note, these studies about the enhancement of graft-versus-tumor effects and the reduction of GVHD by means of combined DLI/trAb application was thoroughly performed upon administration of C57 splenocytes with or without pretreatment of anti-EpCAM x anti-CD3 trAb BiLu in (BALB x C57BL/6) F_1_ H-2^d/b^ mice that were previously inoculated with a lethal dose of B16-EpCAM melanoma cells. Importantly, the C57 spleen cells were syngeneic to the B16-EpCAM tumor cells, but haploidentically mismatched to host cells. Treatment with BiLu protected recipient mice from the alloreactive C57 splenocytes, as GVHD-related death was observed in only 20% of mice whereas almost all mice inoculated with naive C57 splenocytes w/o BiLu pretreatment died of GVHD [29]. In total 50% of all treated mice were disease-free in this experimental settings. BiLu treatment w/o cell therapy also had a substantial anti-tumor effect, as a similar proportion of treated mice were disease-free under these conditions [29]. Thus, future trials could also address the feasibility to pass on DLI in relevant clinical settings. Here, this clinical study with a combined trAb/DLI treatment might provide further insights into the capacity of this therapeutic regime to deplete malignant B cells, activate the immune system and induce secondary cell-mediated immunity.

## Trial status

The study is currently recruiting patients.

## Competing interests

The authors Raymund Buhmann, Hans-Jochem Kolb and Christian Peschel declare, that they have no competing interest. Horst Lindhofer is CEO and founder of the company Trion Pharma GmbH providing the antibody FBTA05 (Bi20) for the clinical trial. Juergen Hess is responsible for scientific affairs at Trion Pharma GmbH and declares no competing interest. Michael Stanglmaier is head of clinical research / hematological malignancies of the company Trion Research GmbH declaring no competing interest.

## Authors’ contributions

CP is principal investigator (as stipulated by the German Drug Law). RB, MS, JH and HJK designed and planned the FBTA05 trial and drafted the manuscript. CP and HL made contributions to the conception and design of the trial and critically revised the manuscript for important intellectual content. All authors read and approved the final manuscript.

## References

[B1] Grigg A Ritchie D Graft-versus-lymphoma effects: clinical review, policy proposals, and immunobiology Biol Blood Marrow Transplant 2004 10 579 590 10.1016/j.bbmt.2004.05.008 15319770

[B2] Butcher BW Collins RH Jr The graft-versus-lymphoma effect: clinical review and future opportunities Bone Marrow Transplant 2005 36 1 17 10.1038/sj.bmt.1705008 15895112

[B3] Ringden O Karlsson H Olsson R Omazic B Uhlin M The allogeneic graft-versus-cancer effect Br J Haematol 2009 147 614 633 10.1111/j.1365-2141.2009.07886.x 19735262

[B4] Bierman PJ Sweetenham JW Loberiza FR Jr Taghipour G Lazarus HM Rizzo JD Schmitz N van Besien K Vose JM Horowitz M Goldstone A Syngeneic hematopoietic stem-cell transplantation for non-Hodgkin's lymphoma: a comparison with allogeneic and autologous transplantation–The Lymphoma Working Committee of the International Bone Marrow Transplant Registry and the European Group for Blood and Marrow Transplantation J Clin Oncol 2003 21 3744 3753 10.1200/JCO.2003.08.054 12963703

[B5] Khouri IF Lee MS Saliba RM Jun G Fayad L Younes A Pro B Acholonu S McLaughlin P Katz RL Champlin RE Nonablative allogeneic stem-cell transplantation for advanced/recurrent mantle-cell lymphoma J Clin Oncol 2003 21 4407 4412 10.1200/JCO.2003.05.501 14645431

[B6] Escalon MP Champlin RE Saliba RM Acholonu SA Hosing C Fayad L Giralt S Ueno NT Maadani F Pro B Nonmyeloablative allogeneic hematopoietic transplantation: a promising salvage therapy for patients with non-Hodgkin's lymphoma whose disease has failed a prior autologous transplantation J Clin Oncol 2004 22 2419 2423 10.1200/JCO.2004.09.092 15197204

[B7] Khouri IF McLaughlin P Saliba RM Hosing C Korbling M Lee MS Medeiros LJ Fayad L Samaniego F Alousi A Eight-year experience with allogeneic stem cell transplantation for relapsed follicular lymphoma after nonmyeloablative conditioning with fludarabine, cyclophosphamide, and rituximab Blood 2008 111 5530 5536 10.1182/blood-2008-01-136242 18411419 PMC4624452

[B8] Maloney DG Grillo-Lopez AJ White CA Bodkin D Schilder RJ Neidhart JA Janakiraman N Foon KA Liles TM Dallaire BK IDEC-C2B8 (Rituximab) anti-CD20 monoclonal antibody therapy in patients with relapsed low-grade non-Hodgkin's lymphoma Blood 1997 90 2188 2195 9310469

[B9] McLaughlin P Grillo-Lopez AJ Link BK Levy R Czuczman MS Williams ME Heyman MR Bence-Bruckler I White CA Cabanillas F Rituximab chimeric anti-CD20 monoclonal antibody therapy for relapsed indolent lymphoma: half of patients respond to a four-dose treatment program J Clin Oncol 1998 16 2825 2833 9704735 10.1200/JCO.1998.16.8.2825

[B10] Foran JM Gupta RK Cunningham D Popescu RA Goldstone AH Sweetenham JW Pettengell R Johnson PW Bessell E Hancock B A UK multicentre phase II study of rituximab (chimaeric anti-CD20 monoclonal antibody) in patients with follicular lymphoma, with PCR monitoring of molecular response Br J Haematol 2000 109 81 88 10.1046/j.1365-2141.2000.01965.x 10848785

[B11] Smith MR Rituximab (monoclonal anti-CD20 antibody): mechanisms of action and resistance Oncogene 2003 22 7359 7368 10.1038/sj.onc.1206939 14576843

[B12] Stanglmaier M Faltin M Ruf P Bodenhausen A Schroder P Lindhofer H Bi20 (FBTA05), a novel trifunctional bispecific antibody (anti-CD20 x anti-CD3), mediates efficient killing of B-cell lymphoma cells even with very low CD20 expression levels Int J Cancer 2008 123 1181 1189 10.1002/ijc.23626 18546289

[B13] Ruf P Lindhofer H Induction of a long-lasting antitumor immunity by a trifunctional bispecific antibody Blood 2001 98 2526 2534 10.1182/blood.V98.8.2526 11588051

[B14] Eissler N Ruf P Mysliwietz J Lindhofer H Mocikat R Trifunctional bispecific antibodies induce tumor-specific T cells and elicit a vaccination effect Cancer Res 2012 72 3958 3966 10.1158/0008-5472.CAN-12-0146 22745368

[B15] Ruf P Schafer B Eissler N Mocikat R Hess J Ploscher M Wosch S Suckstorff I Zehetmeier C Lindhofer H Ganglioside GD2-specific trifunctional surrogate antibody Surek demonstrates therapeutic activity in a mouse melanoma model Journal of translational medicine 2012 10 219 10.1186/1479-5876-10-219 23134699 PMC3543252

[B16] Buhmann R Simoes B Stanglmaier M Yang T Faltin M Bund D Lindhofer H Kolb HJ Immunotherapy of recurrent B-cell malignancies after allo-SCT with Bi20 (FBTA05), a trifunctional anti-CD3 x anti-CD20 antibody and donor lymphocyte infusion Bone Marrow Transplant 2009 43 383 397 10.1038/bmt.2008.323 18850012

[B17] Kiewe P Thiel E Ertumaxomab: a trifunctional antibody for breast cancer treatment Expert opinion on investigational drugs 2008 17 1553 1558 10.1517/13543784.17.10.1553 18808314

[B18] Barrett AJ Battiwalla M Relapse after allogeneic stem cell transplantation Expert review of hematology 2010 3 429 441 10.1586/ehm.10.32 21083034 PMC3426446

[B19] Slavin S Morecki S Weiss L Or R Immunotherapy of hematologic malignancies and metastatic solid tumors in experimental animals and man Crit Rev Oncol Hematol 2003 46 139 163 10.1016/S1040-8428(02)00108-7 12711359

[B20] ICH E6 Harmonised Guideline for Good Clinical Practice International Conference on Harmonisation of Technical Requirements for Registration of Pharmaceuticals for Human Use 1996 Geneva, Switzerland: ICH

[B21] Declaration of Helsinki - Ethical principles for medical research involving human subjects Proceedings of the 59th WMA General Assembly 2008 Seoul, Korea: World Medical Association

[B22] Chames P Baty D Bispecific antibodies for cancer therapy: the light at the end of the tunnel? MAbs 2009 1 539 547 10.4161/mabs.1.6.10015 20073127 PMC2791310

[B23] Hess J Ruf P Lindhofer H Cancer therapy with trifunctional antibodies: linking innate and adaptive immunity Future Oncol 2012 8 73 85 10.2217/fon.11.138 22149036

[B24] Muller D Kontermann RE Bispecific antibodies for cancer immunotherapy: Current perspectives BioDrugs 2010 24 89 98 10.2165/11530960-000000000-00000 20199124

[B25] Russell NH Byrne JL Faulkner RD Gilyead M Das-Gupta EP Haynes AP Donor lymphocyte infusions can result in sustained remissions in patients with residual or relapsed lymphoid malignancy following allogeneic haemopoietic stem cell transplantation Bone Marrow Transplant 2005 36 437 441 10.1038/sj.bmt.1705074 15980879

[B26] Bloor AJ Thomson K Chowdhry N Verfuerth S Ings SJ Chakraverty R Linch DC Goldstone AH Peggs KS Mackinnon S High response rate to donor lymphocyte infusion after allogeneic stem cell transplantation for indolent non-Hodgkin lymphoma Biol Blood Marrow Transplant 2008 14 50 58 18158961 10.1016/j.bbmt.2007.04.013

[B27] Riechelmann H Wiesneth M Schauwecker P Reinhardt P Gronau S Schmitt A Schroen C Atz J Schmitt M Adoptive therapy of head and neck squamous cell carcinoma with antibody coated immune cells: a pilot clinical trial Cancer Immunol Immunother 2007 56 1397 1406 10.1007/s00262-007-0283-6 17273869 PMC11030563

[B28] Morecki S Lindhofer H Yacovlev E Gelfand Y Slavin S Use of trifunctional bispecific antibodies to prevent graft versus host disease induced by allogeneic lymphocytes Blood 2006 107 1564 1569 10.1182/blood-2005-07-2738 16234351

[B29] Morecki S Lindhofer H Yacovlev E Gelfand Y Ruf P Slavin S Induction of long-lasting antitumor immunity by concomitant cell therapy with allogeneic lymphocytes and trifunctional bispecific antibody Exp Hematol 2008 36 997 1003 10.1016/j.exphem.2008.03.005 18495330

